# Recommendations of the National Medical Convention

**Published:** 1847-08

**Authors:** 


*Recommendations of the National Medical Convention.*—We are gratified to see evidences that societies in different States are acting in furtherance of measures of reform recommended by the late Medical Convention.

The *Medico-Chirurgical Faculty of Maryland* have "forbidden the Board of Examiners to issue licenses except to such candidates as come up to the standard indicated," and the preliminary education is to be required from office pupils.

The *Connecticut Medical Society* at its session in May 12th, adop- the following resolution :

*Resolved*, That before any person can be admitted into the office of a Physician, he shall furnish evidence of good moral character, and shall be examined by the Preceptor, and one of the Fellows of the Society ; the examination to be upon subjects of English education, and Greek and Latin languages. If found qualified, he is to receive a certificate to that effect, and to be enrolled as a regular student of medicine.

The following, which we copy from the *Medical News*, will be read with great satisfaction. We do not doubt that Medical schools generally will follow the example of the *University of Pennsylvania*. It was but just to concede to the oldest institution in the country the honor of taking the lead in this measure :—

*Medical Department of the University of Pennsylvania.*—We are authorised to announce that the Medical Faculty of the University of Pennsylvania, in accordance with the recommendation of the recent Medical Convention, have resolved to extend their course of instruction. They propose to commence the regular lectures upon Monday the 18th of October, and to continue them until the last Saturday in March, thus adding four weeks to the previous term, and making the whole course about five months and a half. We understand, however, that it is designed to hold the commencement as usual, about the first of April, so that the candidate will not be detained later than in former years.

Since the foregoing was written, we have received the annual circular of the Medical College of the State of South Carolina, from which we cut the following :

"The Faculty cannot suffer the occasion to pass withou expressing their unqualified approbation of the proceedings of the National Medical Convention, which was recently held in the city of Philadelphia. To the series of Resolutions relating to the subject of "a suitable preliminary education," addressed to the Profession at large —and those especially having reference to "judicious reform," and "a uniform and elevated standard of requirements for the degree of M. D.," addressed to the Medical Schools in particular throughout the United States—they give their cordial approval, and declare with promptitude their willingness to adopt them as soon as it shall appear that the generality of the Schools are prepared to enter upon their practical enforcement.

